# Diurnal variation around an optimum and near‐critically high temperature does not alter the performance of an ectothermic aquatic grazer

**DOI:** 10.1002/ece3.5666

**Published:** 2019-09-26

**Authors:** Tiina Salo, Tabea Kropf, Francis J. Burdon, Otto Seppälä

**Affiliations:** ^1^ Environmental and Marine Biology Åbo Akademi University Turku Finland; ^2^ Department of Ecology, Environment and Plant Sciences Stockholm University Stockholm Sweden; ^3^ Department of Aquatic Ecology Eawag, Swiss Federal Institute of Aquatic Science and Technology Dübendorf Switzerland; ^4^ Institute of Integrative Biology ETH Zürich Zürich Switzerland; ^5^ Department of Aquatic Sciences and Assessment Swedish University of Agricultural Sciences Uppsala Sweden; ^6^ Research Department for Limnology University of Innsbruck Mondsee Austria

**Keywords:** fluctuating temperature, invertebrate, population

## Abstract

The growing threat of global climate change has led to a profusion of studies examining the effects of warming on biota. Despite the potential importance of natural variability such as diurnal temperature fluctuations, most experimental studies on warming are conducted under stable temperatures. Here, we investigated whether the responses of an aquatic invertebrate grazer (*Lymnaea stagnalis*) to an increased average temperature differ when the thermal regime is either constant or fluctuates diurnally. Using thermal response curves for several life‐history and immune defense traits, we first identified the optimum and near‐critically high temperatures that *Lymnaea* potentially experience during summer heat waves. We then exposed individuals that originated from three different populations to these two temperatures under constant or fluctuating thermal conditions. After 7 days, we assessed growth, reproduction, and two immune parameters (phenoloxidase‐like activity and antibacterial activity of hemolymph) from each individual. Exposure to the near‐critically high temperature led to increased growth rates and decreased antibacterial activity of hemolymph compared to the optimum temperature, whilst temperature fluctuations had no effect on these traits. The results indicate that the temperature level per se, rather than the variability in temperature was the main driver altering trait responses in our study species. Forecasting responses in temperature‐related responses remains challenging, due to system‐specific properties that can include intraspecific variation. However, our study indicates that experiments examining the effects of warming using constant temperatures can give similar predictions as studies with fluctuating thermal dynamics, and may thus be useful indicators of responses in nature.

## Graphical abstract

We investigated whether the responses of an aquatic invertebrate grazer to optimal and high average temperatures differ when the thermal regime is either constant or fluctuates diurnally. The results indicate that the temperature level per se, rather than the variability in temperature was the main driver altering trait responses. Our study indicates that experiments examining the effects of warming using constant temperatures can give similar predictions as studies with fluctuating thermal dynamics, and may thus be useful indicators of responses in nature.

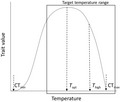

## 1 INTRODUCTION

Temperature is a fundamental driver of many natural processes ranging from metabolism to reproduction and behavior, making the study of organismal performance across thermal ranges an important foundation in our understanding of biology (e.g., John‐Alder, Morin, & Sharon, 1988; Knies, Izem, Supler, Kingsolver, & Burch, 2006; Smith, 1973; Vaughn, 1953). The growing threat of global warming has led to a profusion of studies in the past two decades examining the effects of elevated temperature on biota. Because temperature can be difficult to manipulate and control under natural conditions, field studies that have modified thermal regimes have often had to accept considerable ambient variation and limited replication (Hillebrand, Soininen, & Snoeijs, 2010; Hood et al., 2018; Nelson et al., 2017). Consequently, these challenges mean that most experimental studies assessing the effects of warming are conducted in highly controlled laboratory or outdoor mesocosm settings. Such experiments can be extremely effective by directly testing responses to warming treatments that, for example, simulate future climate scenarios using expected mean or extreme temperatures (IPCC, 2014), but often ignore the natural variability experienced by real ecosystems (Thompson, Beardall, Beringer, Grace, & Sardina, 2013).

**Figure 1:**
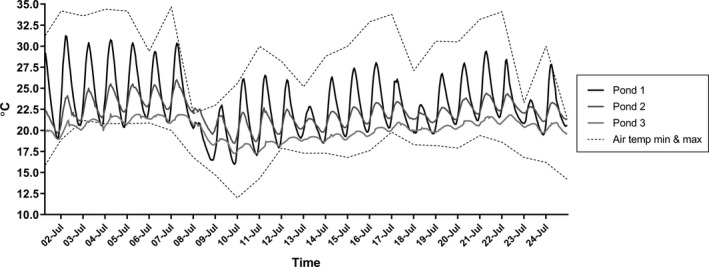
Temperature fluctuation recorded in three natural ponds in Zürich (47°24′10N, 8°35′50E; 47°23′56N, 8°32′57E; and 47°23′58N, 8°32′56E, respectively), Switzerland over 22 days during the 2015 central European heat wave. The dashed lines indicate the maximum and minimum air temperature measured in Zürich (extracted from MeteoSchweiz)

In particular, seasonal or regional trends in temperature are often accompanied by diurnal fluctuations (Bozinovic et al., 2011). Therefore, the difference between the daily maximum and the daily minimum can be large although the magnitude of this difference can vary among locations (Figure 1). Despite such diurnal temperature variation, a great majority of laboratory experiments assess thermal responses of organisms using treatments with a constant temperature. This is because it is easier to expose organisms to constant rather than to fluctuating temperatures, although such a contrivance may affect the conclusions made. Thus, it is imperative to estimate how well constant temperature experiments capture the variation in responses compared to studies with fluctuating temperature. For example, temperature variation can hasten the development of aquatic insects (e.g., Gresens, 1997; Huffaker, 1944; Sweeney & Schnack, 1977). Colinet, Sinclair, Vernon, and Renault (2015) reviewed the few published studies on this topic and found that a fluctuating temperature close to the thermal optimum may improve the organismal performance in insects compared with individuals exposed to constant temperature. Further, a fluctuating temperature close to the thermal extremes may either negatively impact insects due to cumulative damage during exposure or bring thermal refuge from harmfully low or high temperatures (Colinet et al., 2015). Fluctuating thermal dynamics may also yield contrasting responses to high and low temperatures in same organism, for example, simultaneously promoting tolerance to high temperature and reduced tolerance to cold (Salachan & Sørensen, 2017).

Earlier research that compares responses of organisms to constant and fluctuating temperature is largely biased toward terrestrial insects as model organisms (Kutcherov & Lopatina, 2018). Whilst few studies have shown how responses to fluctuating versus constant temperatures may vary between different genders (Fischer, Kölzow, Höltje, & Karl, 2011) and life stages (Salachan & Sørensen, 2017), other studies have observed similar thermal responses across populations (e.g., Fragata et al., 2016; Manenti, Sørensen, & Loeschcke, 2017). Also cryptic phenotypes and their underlying genetic variation, that normally have little or no effect on phenotypic variation in a population, but under extreme conditions can generate heritable traits, may determine a population's ability to adapt to changes in the thermal environment (Paaby & Rockman, 2014). Thus, the role of intraspecific variation among populations in responses to fluctuating versus constant temperatures remains uncertain (cf. Salinas, Irvine, Schertzing, Golden, & Munch, 2019).

**Figure 2:**
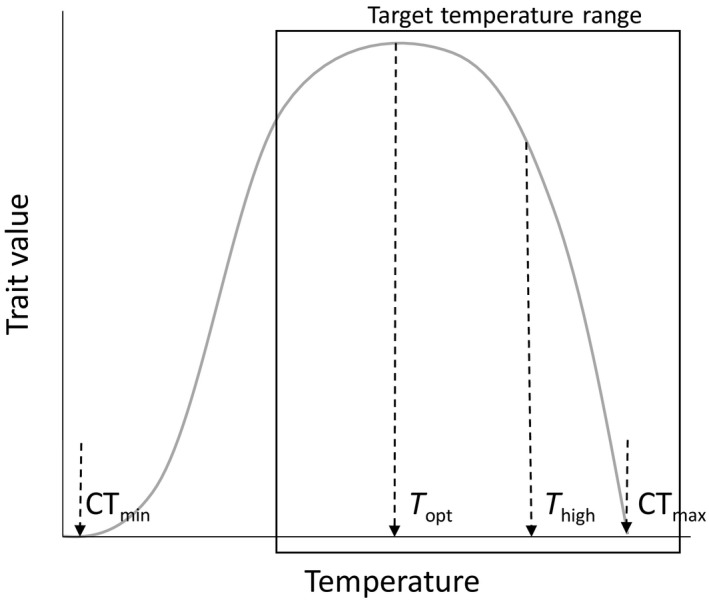
Conceptual thermal performance curve for a hypothetical trait across temperatures ranging from critical minimum (CT_min_) to optimum (*T*_opt_) and to critical maximum (CT_max_). The black box indicates the temperature range targeted in the first experiment to define the optimum temperature (*T*_opt_) and a temperature between the optimum and critical maximum (*T*_high_)

**Figure 3:**
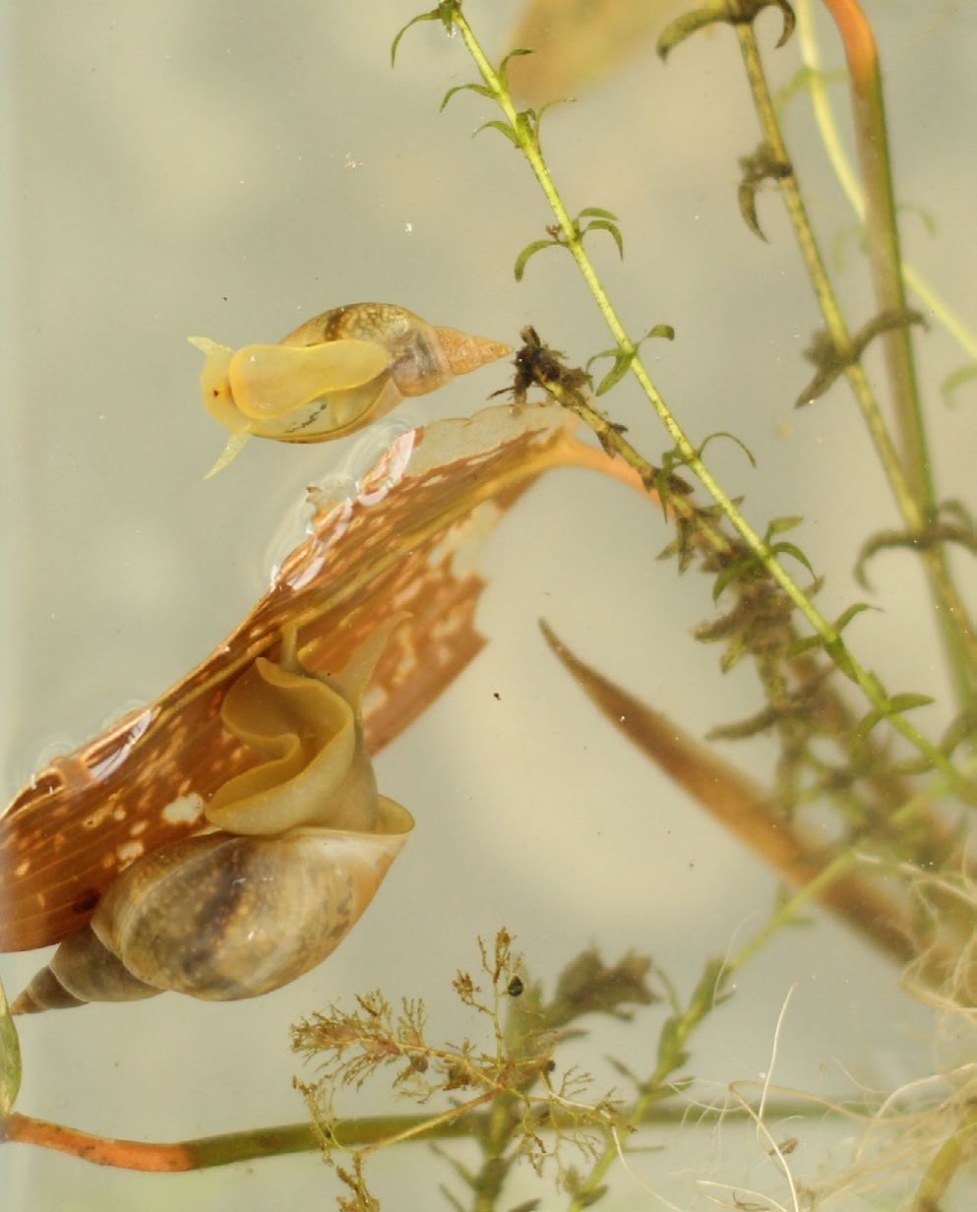
*Lymnaea stagnalis*, the model organism. Photo by A. Taddei

Here, we compared responses to a constant and diurnally fluctuating (±3°C) thermal regime at both “optimum” and “high” (close to the critically high temperature, Figure 2) mean temperatures using the great pond snail, *Lymnaea stagnalis*, as a model organism (Figure 3). We compared the responses of snails that originated from three different populations within the same region. This increased the potential for historical contingencies generating population variability, thus enabling a more general view of thermal responses (constant vs. fluctuating) within this species. *L. stagnalis* is a hermaphroditic pulmonate gastropod (thus, no gender‐based differences), with a wide distribution in stagnant or slowly flowing water bodies in the Northern Hemisphere. It is extensively used as a model organism to investigate the effects of warming (e.g., Leicht, Jokela, & Seppälä, 2013; Salo, Räsänen, Stamm, Burdon, & Seppälä, 2018; Salo, Stamm, Burdon, Räsänen, & Seppälä, 2017; Seppälä & Jokela, 2011) and pollutants (e.g., Coutellec & Lagadic, 2006; Nyman, Schirmer, & Ashauer, 2014; Salo et al., 2018; Salo et al., 2017) on organisms, as well as host‐parasite interactions (e.g., Karvonen, Savolainen, Seppälä, & Valtonen, 2006; Leicht & Seppälä, 2014) and immunology (e.g., Dikkeboom, Knaap, Meuleman, & Sminia, 1985; Seppälä & Leicht, 2013). In this species, exposure to high temperatures increases several organismal process rates (Salo et al., 2018), which leads to increased growth rate and reproductive output with a temporal threshold (1 week), after which the reproductive rate is reduced (Leicht et al., 2013). High temperatures also reduce snail immune defense (Leicht et al., 2013; Salo et al., 2017; Seppälä & Jokela, 2011), which increases their susceptibility to trematode parasites (Leicht & Seppälä, 2014). However, these earlier studies on the effects of warming have all used experimental treatments with constant temperatures. Following Colinet et al. (2015), we made two main predictions regarding snail performance under constant or fluctuating temperatures. Firstly, we expected that temperature fluctuations mimicking natural diurnal patterns around the optimum temperature would be either neutral or beneficial for snails (i.e., no impact or increased growth rate, reproductive output and immune defense compared to constant temperature conditions at the same average temperature). Secondly, we expected that the same fluctuations close to the critically high temperature would be harmful (i.e., reduced trait values compared to conditions with constant temperature at the same average temperature). Better understanding of how this realistic source of uncertainty affects organismal responses to temperature helps address the potential risks in conclusions based on studies using constant temperature treatments.

## 2 MATERIAL AND METHODS

The study was carried out in two phases. In the first phase, we determined the thermal performance curves ranging from medium to high temperature for four traits (reproduction, growth, and two immune parameters) to select “optimum” and “close to critically high” average temperatures (Figure 2) to be used in the second phase of the study. In the second phase, we then assessed whether snails' responses to these two average temperatures depended on whether the temperature was constant or fluctuating (±3°C).

### 2.1 Phase 1: thermal performance curves

Typically, thermal performance curves show the highest performance (signifying optimum temperature) at an intermediate temperature and reduced performance at temperatures below and above it (Figure 2). As we were interested in the effects of warming, we examined snail phenotype at temperatures ranging from mid to high (Figure 2): 15, 18, 21, 24, 27, 30, and 33°C. This range covers the optimum temperature for the growth of juvenile *L. stagnalis* snails (Vaughn, 1953) and the typical temperatures in ponds in the study region (Zürich, Switzerland; T. Salo unpublished data). To estimate a general population‐independent thermal response curve, we used a laboratory stock population created by mating individuals that originated from seven different donor populations in Northern Switzerland (Langeloh, Behrmann‐Godel, & Seppälä, 2017). A total of 140 adult individuals from the fifth generation of this “mixed” population (shell length: 28.7 ± 0.3 mm, average ± *SE*) were placed in 200 ml glass containers filled with aged tap water. The snails were allowed to acclimate for 18 days to 15°C in a climate chamber, after which the temperature was increased by placing the containers holding the snail into water baths with the target water temperature (20 snails per temperature). The target temperatures in the water baths were reached by using EHEIM aquarium heaters (100 W) and circulation pumps to distribute the temperature evenly. Water in the experimental containers was changed every second day, and all individuals were fed ad libitum with fresh lettuce.

As traits may differ in their thermal responses, we assessed multiple traits to generalize across single trait thermal performance curves and to gain more reliable estimates for “optimum” and “high” temperatures. Survival was inspected every second day and dead individuals were removed from the containers. After 10 days, growth, reproduction, and two immune parameters were assessed for each surviving individual (see the Section 2.3).

### 2.2 Phase 2: fluctuating versus constant optimum and high temperatures

**Figure 4:**
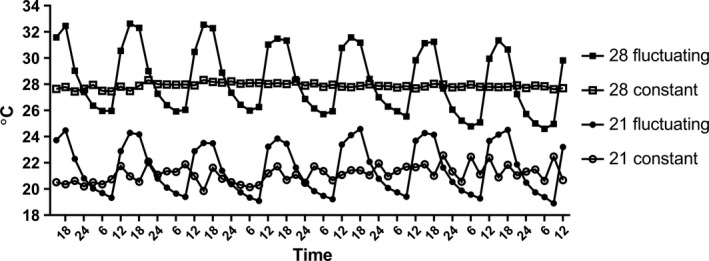
Temperatures during the experiment. Filled symbols indicate the fluctuating temperature treatments and open symbols constant temperature treatments. Squared and circle symbols indicate the daily average temperature of 28°C and 21°C, respectively

Two target average temperatures, “optimum” (21°C) and “high” (28°C), were selected upon estimating the above‐mentioned thermal performance curves. The temperature with the highest snail performance was selected as “optimum” and a temperature close but still below the critically high temperature was selected as “high” (see Figure 2, Sections 2.4 and 3). The temperature measurements recorded during summer 2015 with Onset HOBO temperature loggers in three shallow (<2 m, surface area ca 9–50 m^2^ depending on the pond) ponds in the study region (Northern Switzerland) illustrate how temperature typically follows a diurnal pattern with a maximum in the late afternoon and a minimum in the early morning (Figure 1). Further, the amplitude of thermal variation differs among ponds even within close proximity (Figure 1). We mimicked this diurnal variation in the fluctuating temperature treatments and chose an intermediate ±3°C daily variation for the temperature. We used a full factorial experimental design with four treatment combinations: optimum constant, optimum fluctuating, high constant and high fluctuating temperature (Figure 4), with the same average temperature in the fluctuating and constant temperature treatments at each temperature level. In total eight water baths were established, leading to two water baths per treatment combination. The target temperatures were reached by placing aquarium heaters (EHEIM, 100–300 W) together with a water pump to a water bath. Fluctuations were obtained by switching one heater per bath on and off at specific times in the morning and in the afternoon, respectively, allowing the water to heat and cool as planned. An additional heater was installed in each water bath to ensure a stable minimum target temperature. The temperature in each water bath was quantified every 10 min using Onset HOBO temperature loggers. The final temperature profiles in the different treatments were 21.1 ± 1.4°C, 21.6 ± 2.8°C, 27.9 ± 0.9°C, and 28.3 ± 3.5°C, respectively (Figure 4).

As populations may differ in their thermal responses and we aimed to study general patterns under exposure to constant and fluctuating temperatures, we used individuals from three laboratory stock populations that originated from three different locations in Northern Switzerland: Irchel (47°23′57N, 8°32′57 E), Ittingen (47°34′59N, 8°51′52E), and Zürichberg (47°23′32N, 8°33′52E). All three ponds were shallow (depth < 2 m), and the surface area ranged between ca 9–50 m^2^. These three locations were also included in the seven donor locations for the “mixed population” that was used in the first phase of the study (see above). Although Figure 1 represents thermal fluctuations in ponds from the same geographic region with similar depth and size profiles, these are not the same as the three donor locations used to source snail populations. All populations used in the experiments were kept for ca three generations in 1,000 L tanks with constant flow through and aeration. A total of 108 adult snails (shell length: 29.1 ± 0.24, 28.6 ± 0.23 and 27.8 ± 0.15 mm, for Irchel, Ittingen, and Zürichberg, respectively) from each population were placed individually in 200 ml containers and allowed to acclimate in 21°C water baths for 5 days before the experiment. After that, the individuals were randomly assigned to different temperature treatments so that the number of individuals from each population in each treatment combination and water bath was approximately the same (*n* = 27 per population per treatment combination and *n* = 13 or 14 per population per water bath). The transfer to the target water baths took place in containers filled with 21°C water to allow for a gradual change in water temperature. After seven days, growth, reproduction, and immune defense parameters were measured (see the Section 2.3).

### 2.3 Response variables

The growth of individuals was quantified by measuring their shell length at the beginning and the end of the experiment to the closest 0.1 mm. These measurements were then used to calculate the specific growth rate according to Equation (1),

(1) $$\text{Specific}\;\text{growth}\;\text{rate}=\frac{\left(\ln S_{2}-\ln S_{1}\right)}{\Delta t}$$

where *S*_1_ and *S*_2_ are lengths at the beginning and at the end of the experiment, respectively, and *t* is the time between the measurements in days (Seppälä, Karvonen, Kuosa, Haataja, & Jokela, 2013).

To measure snail reproductive output, all egg clutches oviposited by the snails were collected and counted. To do this egg clutches were photographed on a light table, and the number of embryos in each image was then counted by using cell counter function in Image‐J (Salo et al., 2017). The total number of embryos produced during the treatments (excluding acclimation period) was used as a measure for reproductive output.

The immune defense parameters phenoloxidase (PO)‐like activity and antibacterial activity were measured using hemolymph collected from each snail at the end of the experiment. In invertebrates, including molluscs, phenoloxidase enzymes form part of the defenses against eukaryotic pathogens (Cerenius & Söderhäll, 2004), while humoral antibacterial enzymes are used against microbial infections (Imler & Bulet, 2005; Leicht et al., 2013). Each snail was blot dried and its foot was gently tapped using a pipette tip until it retreated into the shell simultaneously releasing hemolymph through the hemal pore (Sminia, 1981). Ten µl of hemolymph was mixed with 100 µl of PBS buffer and 100 µl pure hemolymph were collected for PO‐like and antibacterial activity analyses, respectively. The samples were frozen in liquid nitrogen, stored at −80°C and analyzed using l‐Dopa and *Escherichia coli* assays for PO‐like activity and antibacterial activity according to Leicht et al. (2013). Briefly, the enzyme PO oxidizes l‐Dopa causing an increase in optical density of the solution, while antibacterial enzymes in the hemolymph destroy lyophilized *E. coli* cells that decrease optical density. The changes in optical density were measured spectrophotometrically (SpectraMax 190, Molecular Devices).

### 2.4 Statistical analyses

The thermal response curves were analyzed by fitting the data with a polynomial regression (*y* = *ax* ^2^ + *bx* + *c*) for each examined trait (in R 3.5.3). The estimated highest performance (*y*_max_) was then derived by using the root of the first derivative of the function. Because all individuals maintained at 33°C died during the experiment, snail growth, and immune activity could not be assessed in this treatment. Thus, data from 33°C were included only in the assessment of reproductive output.

The effect of fluctuating versus constant temperature on different snail populations was analyzed using permutational multivariate analyses of variance (PERMANOVA + 1.0.3 package in PRIMER 6.1.13). Average temperature (“optimum”, “high”) and variation in temperature (“constant”, “fluctuating”) were considered as fixed factors, while population (“Irchel”, “Ittingen”, “Zürichberg”) and water bath (1–8) were considered as random factors. Water bath was nested under treatment combinations (i.e., average temperature and variation in temperature) to explain variation in the data that could arise from maintaining the snails in different water baths. As many of the responses can be size‐dependent (e.g., growth and reproduction), we used the geometric mean of body size (mm) of each individual as a covariate in the analyses. Analyses were started with a multivariate analysis including all four response variables and followed by univariate analyses on each individual response variable. Prior to the analyses, each variable was normalized by subtracting the mean across all samples from each data point and then dividing by the standard deviation of that variable. The resemblance matrixes were based on Euclidean distance. All analyses were run 9,999 times using type I SS.

## 3 RESULTS

### 3.1 Phase 1: thermal performance curves

**Figure 5:**
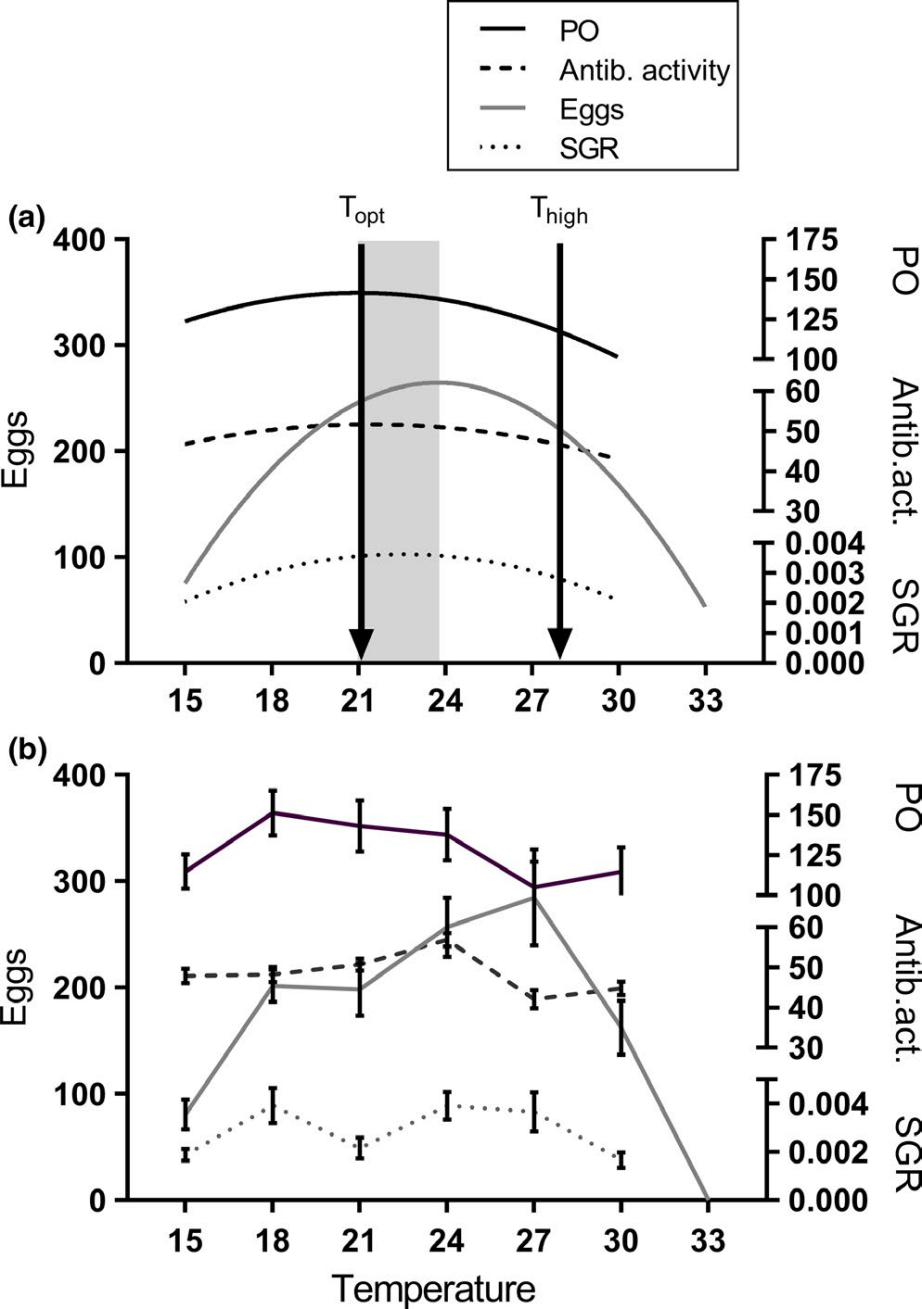
(a) Quadratic regressions for *Lymnaea stagnalis* traits at temperatures ranging from 15 to 33°C and (b) the observed trait values at these temperatures. The solid gray line indicates reproductive rate (left *y*‐xis), solid black line phenoloxidase‐like activity (top right *y*‐axis), dashed line antibacterial activity (midright *y*‐axis), and dotted line specific growth rate (lower right *y*‐axis). The error bars indicate *SE*. Lack of data for 33°C is due to high mortality during the experiment in this treatment. The gray area in (a) indicates the optimum temperature window (21.1–23.8°C) based on the estimated maximum y‐values of the regressions. The arrows indicate the “optimum” (*T*_opt_) and “close to critically high” (*T*_high_) temperatures selected for the fluctuating temperature experiment

Survival was high at temperatures ≤27°C (95% at 15–18°C, 90% at 21°C, 100% at 24°C, 90% at 27°C), decreased to 80% at 30°C and to 0% (100% mortality) at 33°C. The thermal performance curves for growth, reproduction, PO‐like activity, and antibacterial activity of snails showed slightly different estimated optimal temperatures (Table 1, Figure 5a). Reproductive output, specific growth rate, and antibacterial activity were estimated to reach their maximums at 23.8°C, 22.4°C, and 21.4°C, respectively. The quadratic regression for PO‐like activity of snail hemolymph was nonsignificant, but peaked at 21.0°C.

**Table 1:** Estimates from quadratic regressions for number of eggs, specific growth rate, PO‐like activity, and antibacterial activity

Trait	*a*	*b*	*c*	*t*_opt_	*R* ^2^	*F_df_*	*p*
No of eggs	−2.46	116.93	−1,125.00	23.8	0.224	*F*_2,117_ = 18.210	<.001
SGR	0.00	0.00	−0.01	22.4	0.045	*F*_2,106_ = 3.522	.033
**PO (ns)**	**−0.48**	**20.30**	**−72.09**	**21.0**	**0.020**	*F*_2,104_ = **2.062**	**.132**
Antib	−0.12	5.08	−2.72	21.4	0.087	*F*_2,100_ = 5.889	.004

Note

*a*, *b*, and *c* are estimates for the quadratic equation *y* = *ax* ^2^ + *bx* + *c*, *t*_opt_ indicates the estimated optimum temperature. The nonsignificant regression is indicated by bold.

As all traits reached their estimated maximum at 21.0–23.8°C, 21°C was chosen as the “optimum” average temperature for the following phase of the study, allowing the fluctuating temperature (±3°C) to be within the window of maximum values for the different traits. The “high” temperature for the following phase was set to 28°C. This temperature placed the 28 ± 3°C fluctuations within the decreasing slope for most of the traits without reaching the lethal 33°C. This estimated “optimum” temperature was close to the observed maximum performance detected at different experimental temperatures for different traits: Reproductive output was highest at 27°C, antibacterial activity at 24°C, and PO‐like activity and specific growth rate at 18°C, with an average of 21.8°C across the traits (Figure 5b).

### 3.2 Phase 2: fluctuating versus constant optimum and high temperatures

**Figure 6:**
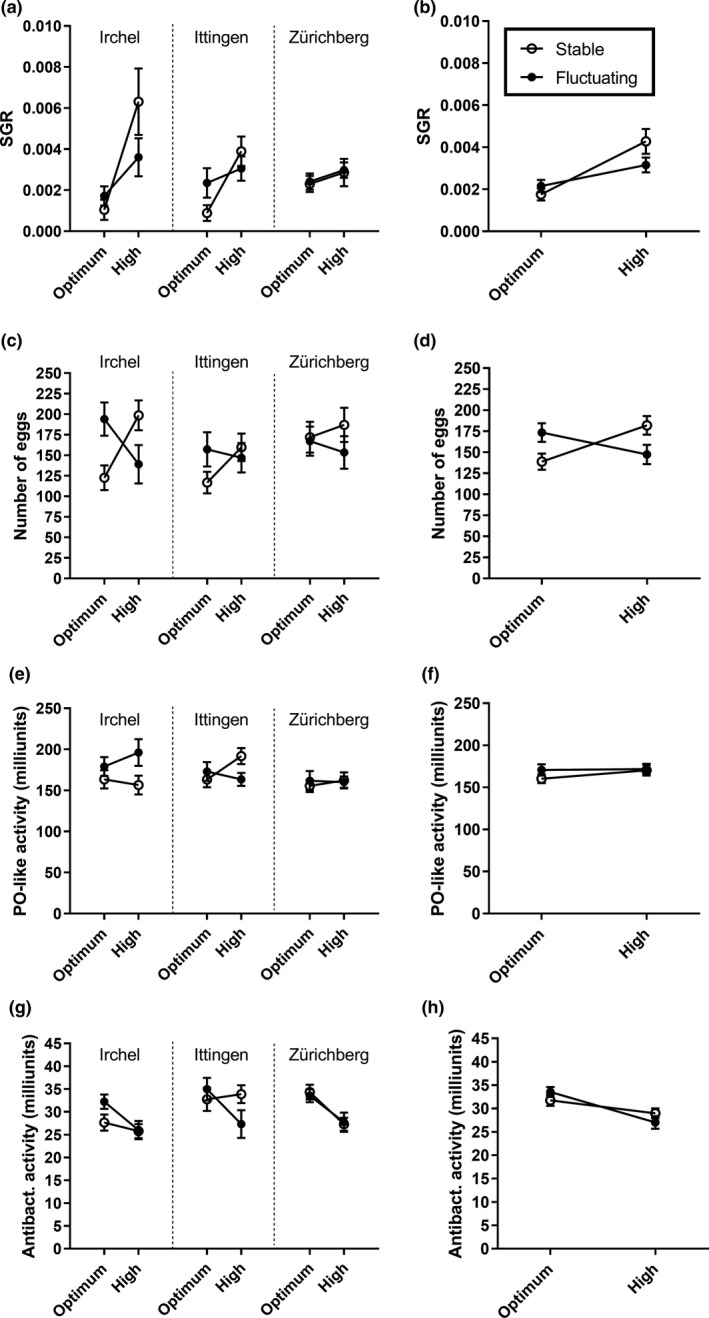
Average ± *SE* for specific growth rate (a, b), number of eggs (c, d), PO‐like activity (e, f), and antibacterial activity (g, h) in combination of different temperatures (optimum, high: *x*‐axis) and variation (constant and fluctuating: open and closed symbols, respectively) for three different populations (left panel; Irchel, Ittingen, Zürichberg). The figures on the right panel illustrate the general responses across populations

The multivariate analysis (PERMANOVA) showed that the average temperature, not temperature fluctuations or their interaction, affected snail performance (Table 2). There was, however, an interactive effect of population and temperature fluctuation on the multivariate phenotype (Table 2). When the responses of different traits were analyzed separately (Figure 6), growth rate increased and antibacterial activity decreased at high temperature (Table 3, Figure 6b,h). Whether individuals were exposed to constant or fluctuating temperature had no impact on the responses (Figure 6b,h). Populations differed in their growth rates at the tested average temperature levels (Table 3, Figure 6a). Further, the PO‐like activity was interactively dependent on population and fluctuating temperature (Table 3, Figure 6c). Reproductive output and PO‐like activity increased with size of individuals (Table 3).

**Table 2:** Results from multivariate permutational analysis of covariance

Source	*df*	MS	Pseudo‐*F*	*p*(perm)	*η* ^2^
Covariate	1	17.428	4.744	**.003**	1.7
Average temp.	1	48.851	2.643	**.028**	4.7
Temp. fluct.	1	3.888	0.337	.636	0.4
Population	2	4.927	1.398	.194	0.9
AT × TF	1	15.703	0.932	.178	1.5
AT × P	2	4.994	1.419	.183	1.0
TF × P	2	7.194	2.043	**.037**	1.4
AT × TF × P	2	5.785	1.661	.103	1.1
Water bath (AT × TF)	4	14.668	4.208	**<.001**	5.6
Residual	244	3.482			81.7

Note

Data includes specific growth rate, number of eggs, PO‐like activity, and antibacterial activity. Covariate is based on size of individuals. Bolded values indicate significant results.

Abbreviations: AT, average temperature; P, population; TF, temperature fluctuation.

**Table 3:** Results from univariate permutational analysis of covariance for specific growth rate, number of eggs, PO‐like activity, and antibacterial activity

Source	*df*	MS	Pseudo‐*F*	*p*(perm)	*η* ^2^
**Specific growth rate**					
Covariate	1	1.208	1.339	.229	0.5
Average temp.	1	18.029	3.594	**.043**	6.9
Temp. fluct.	1	0.491	0.517	.362	0.2
Population	2	0.721	0.806	.451	0.6
AT × TF	1	3.577	1.854	.107	1.4
AT × P	2	3.694	4.130	**.018**	2.8
TF × P	2	1.162	1.299	.274	0.9
AT × TF × P	2	0.880	0.986	.375	0.7
Water bath (AT × TF)	4	1.516	1.699	.151	2.3
Residual	244	0.892			83.7
**Number of eggs**					
Covariate	1	8.947	9.630	**.002**	3.4
Average temp.	1	1.076	0.400	.411	0.4
Temp. fluct.	1	0.017	0.172	.608	0.0
Population	2	2.119	2.420	.095	1.6
AT × TF	1	10.791	1.662	.126	4.2
AT × P	2	0.272	0.311	.743	0.2
TF × P	2	0.549	0.627	.545	0.4
AT × TF × P	2	2.357	2.736	.068	1.8
Water bath (AT × TF)	4	4.592	5.315	**.001**	7.1
Residual	244	0.862			80.9
**PO‐like activity**					
Covariate	1	7.249	7.620	**.010**	2.8
Average temp.	1	0.961	2.038	.100	0.4
Temp. fluct.	1	1.264	0.511	.358	0.5
Population	2	1.162	1.218	.297	0.9
AT × TF	1	0.153	0.392	.425	0.1
AT × P	2	0.288	0.302	.736	0.2
TF × P	2	3.689	3.866	**.023**	2.8
AT × TF × P	2	2.193	2.296	.104	1.7
Water bath (AT × TF)	4	0.654	0.683	.599	1.0
Residual	244	0.955			89.7
**Antibacterial activity**					
Covariate	1	0.025	0.028	.866	0.0
Average temp.	1	28.786	3.395	**.048**	11.1
Temp. fluct.	1	2.116	0.297	.491	0.8
Population	2	0.925	1.156	.325	0.7
AT × TF	1	1.183	0.237	.546	0.5
AT × P	2	0.740	0.929	.397	0.6
TF × P	2	1.794	2.248	.104	1.4
AT × TF × P	2	0.355	0.460	.625	0.3
Water bath (AT × TF)	4	7.906	10.236	**<.001**	12.2
Residual	244	0.773			72.6

Note

Covariate is based on size of individuals. Bolded values indicate significant results.

Abbreviations: AT, average temperature; P, population; TF, temperature fluctuation.

## 4 DISCUSSION

Responses of organisms to changing temperature conditions can be alleviated by behavioral and physiological modifications of the phenotype (i.e., plastic responses). These responses are, however, typically examined in experiments where organisms are exposed to constant temperatures without mimicking natural thermal regimes (e.g., diurnal variation). The use of such artificial conditions has been criticized, because it potentially underestimates the effects of warming on organisms (Paaijmans et al., 2013). To compare how responses to constant versus fluctuating temperatures differ in an aquatic grazer species, we exposed three populations of the snail *L. stagnalis* to “optimum” and “high” average temperatures both with and without diurnal fluctuations in temperature. The results indicate that the temperature level per se, rather than the variability in temperature is the main driver altering trait responses in our study organism. The responses to average temperature varied more strongly among the examined populations than between fluctuating and constant temperature conditions.

Our results show that diurnal temperature variation across all populations had no significant independent effects on organismal traits. This contrasted with the results of Bozinovic et al. (2011) showing that thermal variation enhanced the rate of population growth at a low average temperature but depressed this rate at a high average temperature. Theoretically, whether or not fluctuating and constant temperatures with the same average generate different or similar responses in organisms depends on (a) the shape of the species' thermal response curve, (b) at which average temperature the fluctuations take place as well as (c) the magnitude of temperature fluctuation (Colinet et al., 2015). Other factors that might further influence responses include the duration and frequency (i.e., predictability) of temperature fluctuations (Manenti, Sørensen, Moghadam, & Loeschcke, 2014).

Thermal response curves for the studied traits indicate that our study organism has a wide tolerance for temperatures on the warmer side of the temperature optimum. This generalist‐like functioning over a large range of temperatures could potentially explain the lack of responses to fluctuating temperatures in comparison to constant temperatures. Such a response could be expected in our study organism, given its wide distribution and preference for standing water habitats. In contrast, aquatic ectotherms with a more asymmetric temperature envelope could be more susceptible to thermal variation. For example, semi‐voltine aquatic insects that emerge in spring (e.g., some Plecoptera and Ephemeroptera) have short‐life cycles which exposes nymphs to lower and less variable temperatures than other longer‐lived species, resulting in a more asymmetric thermal niche with a lower critical thermal maximum (Ernst, Beitinger, & Stewart, 1984). Nonetheless, the daily maximum temperature in our treatment with high fluctuating temperature (28 ± 3°C) did get close to the upper thermal limit of our study organism (100% mortality at 33°C in the thermal performance curve experiment).

Whether temperature fluctuations do or do not differ from exposure to constant temperature with the same mean temperature can depend on whether the fluctuating temperature reaches over linear, convex or concave part of a thermal response curve (Colinet et al., 2015). At temperatures with linear change, fluctuations should not affect the response, while during concave or convex thermal response may increase and decrease the response in relation to constant temperature, respectively (Paaijmans et al., 2013). Hence, the consistently high performance independent of fluctuations at high temperature could indicate somewhat linear response to high temperatures in our model organism. Furthermore, in a study involving a mosquito model, thermal variation strongly reduced the performance at near‐critically high temperatures (Paaijmans et al., 2013). Thus, the similar responses to constant and varying temperature at the high temperature treatment used in our study suggest that our study species uses the thermal refuge from high temperatures (i.e., cooling periods) for recovery in order to preserve individual performance. High temperatures are thus likely to require more cumulative exposure (i.e., longer duration) to adversely impact our study species.

The discrepancies between responses to fluctuating and constant temperatures have been suggested to depend on how temperature‐sensitive the respective trait is (Colinet et al., 2015). While we assessed several traits, only reproductive output showed a slight (nonsignificant) tendency toward our hypothesis of temperature fluctuations improving reproductive output at optimum temperature but decreasing the output when exposed to high temperature. This could reflect either that (a) reproduction does not gain from the thermal refuge to the same degree as other traits, or that (b) the fluctuating temperature provides enough thermal refuge to prevent terminal investment (Williams, 1966).

Just as acclimation can increase an individual's thermal tolerance (Colinet et al., 2015; Marshall, Brahim, Mustapha, Dong, & Sinclair, 2018), natural selection should alter thermal responses in populations over contemporary evolutionary timescales (Merilä & Hendry, 2014). For instance, Leicht, Seppälä, and Seppälä (2017) observed high family‐level trait variation in *L. stagnalis* at different temperatures suggesting some evolutionary potential in response to warming in our model organism. However, the evidence still remains weak, with numerous examples contradicting these predictions (e.g., Fragata et al., 2016; van Heerwaarden, Lee, Overgaard, & Sgrò, 2014; Manenti et al., 2017). For example, both allopatric and laboratory‐adapted populations of the terrestrial dipteran insect *Drosophila melanogaster* demonstrated highly conserved responses to fluctuating temperatures, indicating that short‐term evolutionary responses may be the exception, and not the rule in organismal responses to environmental heterogeneity (Manenti et al., 2017). Some potential factors explaining this discrepancy may be the levels of standing genetic variation and presence of cryptic phenotypes in populations (Salinas et al., 2019), and in aquatic environments, rising or extreme temperatures may also be exacerbated by an associated decline in oxygen availability, thus strengthening the selection pressure on aquatic ectotherms (Hoffmann, Chown, & Clusella‐Trullas, 2013). In our experiment, the differences between populations explained more of the variation in trait responses compared to whether organisms were exposed to constant or fluctuating temperatures. For example, the three populations had differing response patterns for PO‐like activity. Had we conducted the study with only one population, the results could either have supported our hypothesis and shown that temperature fluctuations contribute more to the observed variation (population: Ittingen), or potentially have underestimated the importance of thermal variation (population: Zürichberg). Fischer et al. (2011) observed that within‐species variation due to different genders can explain more of the observed variation in trait responses than whether or not the temperature is fluctuating or constant. Our findings, together with the observations of Fischer et al. (2011), emphasize the importance of accounting for intraspecific variability when assessing how environmental changes may alter organismal responses.

Placing our results in context with the previous studies conducted using insects suggests that it is not self‐evident that diurnal temperature fluctuations are important for organismal performance. Even in organisms where fluctuating and constant temperatures yield in different responses, studies exposing organisms to constant temperature levels may still catch a considerable amount of the responses observed under more variable temperatures. For example, Fischer et al. (2011) observed that increased temperature led to faster growth rates in butterflies and while fluctuating and constant temperatures could result in differing average growth rates, the direction of change compared to the low‐temperature treatment was always independent of the temperature variation. Bernhardt, Sunday, Thompson, and O'Connor (2018) further showed that while thermal performance curves of phytoplankton differ depending on whether they experienced constant or fluctuating temperatures, the former can reliably be used to predict the latter. As long as studies concentrate on relative changes rather than mean trait values per se, and consider that decreased environmental variability may yield more conservative results, constant temperature studies may still give a good estimation for temperature‐induced responses (Fischer et al., 2011).

In conclusion, our study suggests that exposure to fluctuating temperatures does not necessarily yield altered responses when compared to constant temperature treatments. Whilst extrapolating results from temperature experiments conducted in benign laboratory conditions should be done with careful consideration, constant temperatures are a valid proxy when estimating responses to warming.

## CONFLICT OF INTERESTS

We declare we have no competing interests.

## AUTHOR CONTRIBUTIONS

TS, TK, FJB, and OS designed the study; TS and TK conducted the experiments and laboratory analyses; TS analyzed the data and TS, FJB, and OS all contributed to the writing.

## Data Availability

The data are available in Dryad Digital Repository https://doi.org/10.5061/dryad.nc6d5f8.
